# Intra-Abdominal Hypertension and Gastrointestinal Symptoms in Mechanically Ventilated Patients

**DOI:** 10.1155/2011/982507

**Published:** 2011-03-30

**Authors:** Annika Reintam Blaser, Pille Parm, Reet Kitus, Joel Starkopf

**Affiliations:** ^1^Anaesthesiology and Intensive Care Clinic, University of Tartu, Puusepa 8, 51014 Tartu, Estonia; ^2^Anaesthesiology and Intensive Care Clinic, Tartu University Hospital, Puusepa 1A, 51014 Tartu, Estonia

## Abstract

*Background*. We aimed to describe the incidence of intra-abdominal hypertension (IAH) and gastrointestinal (GI) symptoms and related outcome in mechanically ventilated (MV) patients. *Methods*. Intra-abdominal pressure (IAP) and gastric residual volumes were measured at least twice daily. IAH was defined as a mean daily value of IAP ≥ 12 mmHg. *Results*. 398 patients were monitored for all together 2987 days. GI symptom(s) occurred in 80.2% patients. 152 (38.2%) patients developed IAH. Majority (93.4%) of patients with IAH had GI symptoms. The more severe IAH was associated with the higher number of concomitant GI symptoms (*P* < .001). 142 (35.7%) patients developed both IAH and at least one GI symptom at any time in ICU, and in 77 patients they occurred simultaneously on the same day. This subgroup had the highest ICU mortality (21.8%). In contrast, the small group of patients presenting only IAH, but not GI symptoms (10 patients), had no lethal outcome. Three patients (4.4%) died without showing either IAH or GI symptoms. *Conclusions*. GI symptoms and IAH often, but not always, occur together. The patients having IAH solely without developing GI symptoms have rather good outcome.

## 1. Introduction

Intra-abdominal hypertension (IAH) and gastrointestinal (GI) symptoms occur frequently in ICU patients [1–3], and both have adverse impact on outcome [1, 3]. However, their correlations have not been extensively studied. IAP measurement is becoming more and more routine in ICU patients, but its role in progress of critical illness is still not enough clarified. The detrimental consequences of IAH as well as high mortality of abdominal compartment syndrome have been well described in recent years [4, 5]. However, the reasons for the development of IAH as well as the associations with GI function are so far largely speculative. IAH is commonly looked at as a separate syndrome, not directly connected to the function of gastrointestinal system. In our previous study, we combined enteral feeding intolerance (FI) together with the development of IAH into a GI failure score, which showed good performance in predicting ICU mortality [6]. Still, the exact associations between the occurrence of GI symptoms (vomiting, absence of bowel sounds, diarrhoea, etc.) and IAH have not been clarified. We have observed that most of the patients with IAH also present GI symptoms [6], but in which order and importance these entities appear is not identified in previous studies. Whether intra-abdominal pressure (IAP) might be useful as a single surrogate marker for GI dysfunction is not clear. It seems logical to assume that GI dysfunction/failure may be as well a reason as a result of IAH. From another perspective, IAH might be considered as a reason for GI symptoms, but also as a symptom of GI dysfunction.

Intolerance to enteral feeding is not uniformly defined, often including some again not well-defined GI symptoms (vomiting, high gastric residual volume, and abdominal distension) [7]. However, despite the problems in definition and reproducibility, the feeding intolerance (FI) has been suggested as a possible parameter for GI dysfunction/failure [8]. FI is frequent in intensive care, and its prevalence is especially high in patients with IAH [6]. 

The aim of our study was to describe the incidence of IAH and GI symptoms and related outcome in mechanically ventilated (MV) patients.

## 2. Materials and Methods

Consecutive patients admitted to General ICU of Tartu University Hospital from October 2006 to June 2009 and requiring mechanical ventilation for at least 24 hours were included. 

We performed a retrospective analysis of prospectively collected data, based on electronic database. Respective database was developed for epidemiological studies on gastrointestinal function and includes daily data of all the patients admitted to the General ICU of the Tartu University Hospital since 2004. During the study period, the measurements of IAP were applied in all MV patients. The Ethics Committee of the University of Tartu approved the conduction of the study. 

Characteristics of study patients are presented in Table 1. 847 patients were treated for 6673 days in participating unit during the study period. 398 of them were mechanically ventilated for at least 24 hours after admission and had the possibility for IAP measurements. These patients were included in the study and monitored for 2987 days of mechanical ventilation in total.

Data for each ICU day with mechanical ventilation were collected for each patient. IAP was measured intermittently at least every 6 hours in patients with an IAP ≥ 12 mmHg or at least every 12 hours in patients with an IAP < 12 mmHg. IAP was measured via bladder catheter, at end-expiration in supine position, using the revised closed system repeated measurement technique [9]. An instillation volume of 25 mL was used [10]. Mid-axillary line was taken as a zero level for IAP readings. Mean and maximum values of IAP were documented for each day. Maximum of the mean daily values of each patient was used to stratify the patient as IAH or no-IAH patient. Accordingly, the patient was stratified as an IAH patient when the mean IAP on at least one day was 12 mmHg or higher.

The presence or absence of gastrointestinal symptoms was documented daily. Gastric residual volumes were measured at least twice daily by opening of nasogastric tube and allowing passive outflow of gastric content into collection bag for at least 30 minutes per measurement. Metoclopramid was commonly used in case of high gastric residuals.

APACHE II [11] was documented for the first 24 hrs and SOFA [12] score daily.

### 2.1. Definitions

*IAH* was defined as a mean daily value of IAP ≥ 12 mmHg.

*Abdominal compartment syndrome *(*ACS*) was defined as a sustained IAP > 20 mmHg with an onset of a new organ failure [10]. The onset of an organ failure was confirmed by a SOFA subscore rise to 3 points or above.

*Feeding intolerance* was defined as forced withdrawal/reduction of feeding due to vomiting, bowel distension, diarrhoea, or high nasogastric residuals.

For *gastrointestinal symptoms* we used the following institutional definitions.

*Vomiting* was defined as regurgitation of gastric contents in any volume. 

*Gastrointestinal bleeding* was defined as macroscopic presence of blood in gastric contents or stool.

*Absent/abnormal bowel sounds* were defined as absent, pathologically high, or infrequent peristaltics according to the doctor's subjective evaluation.

*Ileus/bowel distension* was documented according to the radiological diagnosis. Radiological diagnosis was made according to the subjective decision of the radiologist, no clear-cut values for bowel diameter were used.

*Diarrhoea* was defined as unformed stool occurring more than 3 times/24 hours.

*High nasogastric aspirate* was defined as gastric residuals more than 500 mL/24 hrs.

*Constipation* was defined as absence of bowel passage for 5 or more days.

### 2.2. Group Assignments

(a) Patients according to the development of GI symptoms and IAH were divided into the following groups

1. no GI symptoms or IAH during the ICU stay,
2. GI symptom(s) occurred, IAH did not,
3. IAH occurred, GI symptom(s) did not,
4. both GI symptom(s) and IAH occurred during the ICU stay (any time, not necessarily on the same day),
  (4.1) GI symptom(s) occurred first, IAH at least one day later,
  (4.2) IAH occurred first, GI symptom(s) at least one day later,
  (4.3) GI symptom(s) and IAH occurred simultaneously on the same day.

(b) ICU days according to the presence or absence of IAH were divided into

1. IAP_mean_ < 12 mmHg,
2. IAP_mean_ ≥ 12 mmHg.

### 2.3. Statistics

Statistical Package for the Social Sciences (Versions 15.0 and 17.0 SPSS Inc., Chicago, Ill, USA) software was used for statistical analysis.

Continuous variables are expressed as mean (SD) and categorical data as number of patients (% of patients). Chi-square test was used for comparisons of two groups (IAH versus no-IAH). Analysis of variance (one-way ANOVA) for continuous variables and Kruskal-Wallis test for categorical variables were used to test the differences between multiple groups.

## 3. Results and Discussion

Characteristics of the study group are presented in Table 1. The case-mix does not include cardiac surgical and neurosurgical patients. 105 (26.5%) patients had gastroenteral pathology on admission, 89 (22.4%) patients were admitted due to primary cardiac or pulmonary disease, and 56 patients (14.1%) were polytrauma patients. Admission diagnoses also included intoxications, nephrological, gynaecological, and orthopaedic pathologies. 144 (36.2%) patients had sepsis on admission. 

At least one GI symptom occurred in 320 (80.4%) study patients. 152 (38.2%) patients suffered from IAH for at least one day during their ICU stay. This is somewhat higher incidence than reported previously [1, 3, 6, 13]. Different inclusion criteria are likely the reason for this discrepancy, as current study included only the patients on mechanical ventilation for at least 24 hours. Mechanical ventilation itself is a known risk factor for IAH [10]. To avoid different pathophysiological conditions in ventilated and spontaneously breathing patients as a confounder, we decided to include only ventilated patients for IAP measurements.

The prevalence of GI symptoms and IAH as well as the ICU mortality and length of stay are presented in Table 2. The study group of 398 patients had mean APACHE II score of 15.7 (7.5) points and ICU mortality of 12.8% (54 patients). ACS occurred in six (1.5%) patients; four of them died. The mortality rates and ICU stay differed significantly between the groups. The small group of patients presenting only IAH, but not GI symptoms, had no lethal outcome.

The prevalence of GI symptoms and IAH according to their appearance order is presented in Table 3. 142 (35.7%) of our patients had both IAH and at least one GI symptom at any time in ICU, and in 77 (19.3%) of them GI symptom(s) and IAH occurred simultaneously. The ICU mortality rate and length of stay were not dependent on whether GI symptoms developed before, after, or simultaneously with IAH.

**Figure 1:**
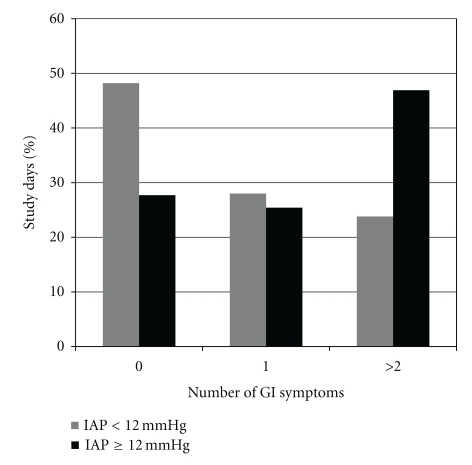
Number of GI symptoms that occurred in comparison of days when IAH was present versus when IAP was normal.

**Figure 2:**
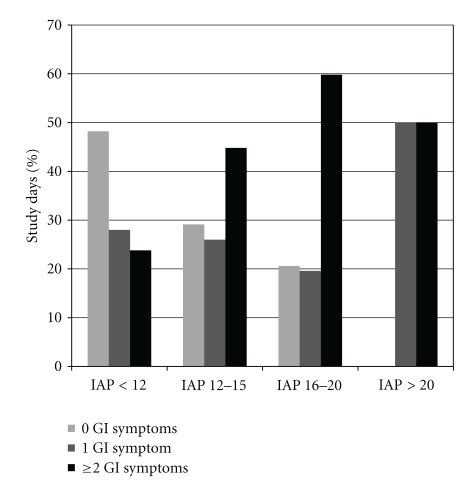
Development of GI symptoms in groups according to the level of IAP.

IAH was observed in 683 (22.9%) study days, while at least one GI symptom occurred on 1680 (56.2%) study days. Enteral feeding was applied in 2176 (72.8%) study days. In 518 days, the enteral feeding was withdrawn/reduced due to GI problems, thus FI occurred in 23.8% of feeding days. The number of days with different GI symptoms and FI according to the presence or absence of IAH is presented in Table 4. The prevalence of GI symptoms in IAH days was higher when compared to the days with normal IAP, *P* < .001 between the groups (Figure 1). 28.3% of IAH-days were free from GI symptoms. The number of GI symptoms was correlated with the severity of IAH, *P* < .001 between the groups (Figure 2). 

These results confirm that not all the patients with gastrointestinal symptoms develop IAH. Moreover, even if most of the patients with IAH also show GI symptom(s), there exists a small group of patients, who suffer from IAH, but do not develop GI symptoms, and in whom the outcome seems to be better. In our study, all these patients (*n* = 10) survived. Based on this, we assume that in some cases IAH may have a modest role in worsening of the progress of critical illness. 

The main limitations of our study are the subjectivity of definitions for GI symptoms and single-centre design. The absence of clear definitions of GI symptoms is not surprising in light of wide range of physiological variability and limited availability of measurement tools of GI function. This clearly complicates the research on GI dysfunction/failure. Even though the clinical importance of auscultation of bowel sounds [14] as well as the need for measuring the gastric residuals [15] has been questioned recently, we believe that the appearance of GI symptoms and GI dysfunction/failure reflect the more severe course of critical illness. The patients with IAH tend to have higher severity scores, but it is not the case in patients with GI symptoms. However, the occurrence of both IAH and GI symptoms is associated with adverse outcome. In our earlier study, we observed similar pattern, showing that simultaneous occurrence of IAH and FI significantly impairs the survival [6]. Diagnosis of FI is usually based on complex clinical evaluation, and there is no single clear-cut symptom or value [7, 8]. It is commonly defined through the occurrence of GI symptoms, which clearly may confuse the diagnostic decisions. For example, first, some GI symptoms are diagnosed subjectively and thereafter another subjective decision about FI is taken. Moreover, the impact of FI on the progress of critical illness might be different depending on the definition and the symptom(s) leading to this diagnosis. Importantly, an attempt to feed is a kind of preconditioning factor to detect FI at all. Therefore, in a current study, we assessed GI symptoms and FI in separate. 

Some of the GI symptoms are most likely the result, while some of them may be the source of IAH. The exact correlations are difficult to determine, and with intermittent measurement of IAP in our study we cannot clear this aspect. We may only speculate on this topic, considering logical patterns of pathophysiology. However, with this theoretical approach most of the symptoms may be looked at as a cause as well as a result of IAH.

Our results might be interpreted in a way that most severe and long-staying patients develop GI symptoms and IAH. But then again, all organ failures might be looked at in a similar way. The main difficulty in assessing the abdominal/gastrointestinal compartment is the lack of terminology and definitions of different symptoms. IAP as the only easily measurable variable is probably not enough to describe the process of critical illness regarding GI dysfunction. Therefore refinement of definitions of GI symptoms and feeding intolerance is of utmost importance. Which variables provide the best reflection of GI function needs to be clarified as well.

## 4. Conclusions

In the present study, we demonstrate that GI symptoms and IAH often, but not always, occur together. The patients having IAH solely without developing GI symptoms have rather good outcome.

## Figures and Tables

**Table 1:** Characteristics of the patients. The values for admission day are presented as mean (SD) if not stated otherwise. Differences between the groups (*P* value) were found using one-way ANOVA for continuous variables and Kruskal-Wallis test for categorical variables.

	Total	no IAH or GI symptoms	IAH or GI symptoms	both IAH and GI symptoms	*P* value
Number of patients (%)	398 (100.0)	68 (17.1)	188 (47.2)	142 (35.7)	
Male gender, number of patients (%)	256 (64.3)	43 (63.2)	116 (61.7)	97 (68.3)	.456
Surgical profile, number of patients (%)	255 (64.1)	28 (41.2)	123 (65.4)	104 (73.2)	.001
Vasopressor/inotrope, number of patients (%)	370 (93.0)	58 (85.3)	176 (93.6)	136 (95.8)	.019
Age, years	57.3 (18.2)	54.0 (20.0)	57.2 (18.5)	59.0 (16.6)	.176
APACHE II, points	15.7 (7.5)	13.3 (7.1)	15.7 (7.6)	16.9 (7.2)	.005
SOFA, points	8.3 (3.7)	6.9 (3.2)	8.1 (3.7)	9.1 (3.6)	<.001
Body mass index, kg/m^2^	27.6 (6.2)	26.7 (5.4)	26.8 (5.4)	29.0 (6.6)	.013
Fluid balance, L/24 h	2.7 (2.9)	2.2 (2.3)	2.5 (2.7)	3.1 (3.3)	.056
Urine output, L/24 h	1.3 (1.2)	1.4 (0.8)	1.4 (1.3)	1.2 (1.1)	.235
Peak inspiratory pressure, cm H_2_O	26.9 (5.8)	24.8 (5.6)	25.8 (5.3)	29.4 (5.8)	<.001
Positive end-expiratory pressure, cm H_2_O	11.0 (3.8)	9.5 (3.6)	10.3 (3.4)	12.8 (3.7)	<.001
Lactate, mmol/L	3.5 (4.4)	2.6 (2.6)	3.5 (4.4)	4.0 (4.9)	.096

**Table 2:** Prevalence of GI symptoms and IAH during the ICU period and respective outcome of the patients. Differences between the groups (*P* value) were found using one-way ANOVA for continuous, and Kruskal-Wallis test for categorical variables.

	Number of pt (%)	ICU days (SD)	Mortality, pt (%)
Total	398 (100.0)	7.6 (9.1)	52 (13.1)
No GI symptom, no IAH	68 (17.1)	2.4 (1.6)	3 (4.4)
GI symptom(s) present, no IAH	178 (44.7)	5.4 (4.9)	18 (10.1)
IAH present, no GI symptom(s)	10 (2.5)	4.1 (1.9)	0 (0.0)
Both IAH and GI symptoms present	142 (35.7)	12.9 (12.3)	31 (21.8)
*P* value		<.001	<.001

**Table 3:** The occurrence of GI symptoms and IAH according to their appearance order in patients presenting both of them during their ICU stay.

	Number of pt (%)	Mortality, pt (%)	ICU days (SD)
GI symptom(s) occurred first, IAH at least one day later	49 (34.5)	9 (18.4)	12.5 (10.8)
IAH occurred first, GI symptom(s) at least one day later	16 (11.3)	2 (12.5)	14.8 (9.9)
IAH and GI symptom(s) occurred simultaneously on the same day	77 (54.2)	20 (26.0)	12.8 (13.6)

Differences in mortality and ICU stay between the groups were not significant (one-way ANOVA and Kruskal-Wallis, resp.).

**Table 4:** The number of days with different GI symptoms according to the presence or absence of IAH at the day of assessment. Data are presented as number of days (%). Chi-square test was used to compare the groups.

	IAP_mean_ < 12 mmHg	IAP_mean_ ≥ 12 mmHg	*P* value
Absent/abnormal bowel sounds	672 (29.2)	346 (50.7)	<.001
Vomiting	651 (28.3)	331 (48.5)	<.001
Gastric residuals >500 mL/day	261 (11.3)	151 (22.1)	<.001
Ileus/bowel distension	117 (5.1)	77 (11.3)	<.001
Constipation	147 (6.4)	43 (6.3)	.510
Diarrhoea	105 (4.6)	45 (6.6)	.023
GI bleeding	42 (1.8)	27 (4.0)	.002
Feeding intolerance	377 (16.4)	169 (24.7)	<.001
